# Identification, cost evaluation, and prioritization of urban traffic congestions and their origin

**DOI:** 10.1038/s41598-022-17404-8

**Published:** 2022-07-29

**Authors:** Nimrod Serok, Shlomo Havlin, Efrat Blumenfeld Lieberthal

**Affiliations:** 1 Azrieli School of Architecture, Tel Aviv University, 6997801 Tel Aviv, Israel; 2 Department of Physics, Bar-Ilan University, 52900 Ramat Gan, Israel

**Keywords:** Physics, Complex networks

## Abstract

The increasing urbanization in the last decades results in significant growth in urban traffic congestion around the world. This leads to enormous time people spent on roads and thus significant money waste and air pollution. Here, we present a novel methodology for identification, cost evaluation, and thus, prioritization of congestion origins, i.e., their bottlenecks. The presented work is based on network analysis of the entire road network from a global point of view. We identify and prioritize traffic bottlenecks based on big data of traffic speed retrieved in near-real-time. Our approach highlights the bottlenecks that have the most significant effect on the global urban traffic flow. We follow the evolution of every traffic congestion in the entire urban network and rank all the congestions, based on the cost they cause (in Vehicle Hours units). We show that the macro-stability that represents the seeming regularity of traffic load both in time and space, overshadows the existence of meso-dynamics, where the bottlenecks that create these congestions usually do not reappear on different days or hours. Thus, our method enables to identify in near-real-time both recurrent and nonrecurrent congestions and their sources.

## Introduction

The twenty-first century can be characterized as the century of the cities. Since 2008, more than 50% of the world’s population lives in urban areas. The increasing urbanization process (with urban population annual growth rate of about 1.8, based on the world bank estimations) is accompanied by the growing usage of vehicles, which leads to a significant increase in traffic congestion in cities around the world^1–4^. The price of congestion is the enormous time spent on roads^5–8^, as well as the increasing fuel consumption, air pollution, and Carbone Dioxide emission^6–8^. Current technological development gave hope that autonomous cars will solve congestion problems as they were expected to reduce the number of private cars by increasing car-sharing. Recent studies, however, suggest that this is not the case^9–12^. There exists extensive work in various disciplines, e.g. urban planning^2^, traffic^13–15^, complexity, and networks^16–22^ that aims at reducing traffic congestion generally, and in urban areas in particular.

The work on identifying traffic bottlenecks has been developed from studying freeway bottlenecks, through urban active bottlenecks, and lately, with the availability of big data—to near real-time identification of traffic bottlenecks. Many studies on freeway identification of traffic bottlenecks suggested evaluating traffic attributes such as flow, speed, or the differences between the travel duration in the road upstream and downstream^23–26^. These methods, however, cannot be applied directly to urban areas due to the different patterns of the road network (e.g. freeways have no intersections of traffic lights) and the travel behavior on it. Hence, other methods were proposed to identify urban bottlenecks^27–34^. For example, Lee et al.^27^ implement a mining model which defined spatiotemporal traffic bottleneck (STB), and thereafter developed three methods to identify STBs in urban networks. Tao et al.^29^, used the Cell Transmission Model (CTM) theory, where the Average Journey Velocity was selected as the measurement of congestion. The availability of big data, retrieved from traffic flow sensors, intrigued new methodologies that use data-driven techniques to identify urban bottlenecks. These works propose new methodologies that may be developed into tools, and implemented in real traffic control systems to relieve congestion and enhance the network performance. Such works employed correlation tests and the implementation of a Dynamic Bayesian network to overcome the lack of data for the entire urban street network (e.g.,^26,30–34^). Ma et al.^33^ combined complex network theory with a user equilibrium model to analyze the evaluation process of traffic bottleneck. Chen et al.^26^ proposed a method to identify traffic bottlenecks by modeling causal relationships between traffic flow sensors located in urban areas. For that, they estimated transfer entropy among these sensors, and constructed causality graphs to identify traffic bottlenecks and discover congestion propagation patterns.

Existing traffic–management solutions that optimize traffic lights address each intersection individually and use bottom-up solutions such as synchronization and slotting to mitigate local congestion. Currently, there is a lacuna in providing an approach that prioritizes specific bottlenecks over the others, in order to optimize the entire road network in near-real-time, as well as to provide a dynamic road pricing that charges each vehicle according to its unique effect on the entire system. As explained by Hamilton: “When a holistic view of traffic management is taken, individual junction efficiencies can suffer to improve the state of the network as a whole… A strategic view of the entire urban network, with improved detection and communication technologies, is required to enter the next evolution of urban traffic control”^35^. Recent work has tried to address the optimization of traffic management solutions, for example, Backfrieder et al.^36^ developed a forecasting algorithm that identifies expected bottlenecks before a traffic jam emerges. It is based on origin–destination data of the vehicles and assumes utilization of vehicle-to-X communication for transmission of contemporary vehicle data such as route source and destination or current position, as well as for the provision of the routing advice for vehicles. Zhao et al.^37^ also focused on urban bottlenecks. They divided the urban road network into a uniform orthogonal grid and identified sources of traffic jams in specific cells. Li et al.^38^ developed a method to identify traffic jams bottlenecks based on the percolation process while using big data, retrieved in real-time, of traffic speeds. They address the issue of how local traffic flows organize collectively into a global urban flow and refer to this process as "traffic percolation". Hamedmoghadam et al.^39^ studied the way heterogeneity of flow demand affects the network flow dynamics under congestion. They used a percolation approach to identify the bottlenecks with the highest impact on the network flows.

In this present work, we developed a methodology to follow in near-real-time and simultaneously the evolution of every traffic congestion in the entire urban network, and rank all the traffic congestions, based on their cost (in vehicle hours (VH) units). We find that non-recurrent traffic congestions dominate the urban traffic and therefore an efficient real-time identification of traffic congestions is critically needed. Our method is innovative as it uses a new strategy, which overcomes the challenges that the near-real-time identification problem poses. Specifically, our method is innovative in two main aspects: (1) It does not aim at predicting the location of future traffic bottlenecks but identifies them as they emerge. Thus, it allows to accurately follow simultaneously all bottlenecks' dynamics and evolution in near real-time even during intervention in the system, for example, by using an adaptive traffic light control system. Moreover, as our method is not based on the identification of historical patterns, it considers all types of bottlenecks—recurrent as well as non-recurrent; and (2) By identifying and prioritizing simultaneously all the bottlenecks in the network, at different times, it highlights which bottlenecks have the most significant effect on the urban traffic flow. These advantages can be implemented in planning transportation systems and reduce urban traffic congestion.

Similar to^30,31,33,38,39^ we address the traffic urban flow as a directed weighted network. We suggest to identify traffic bottlenecks based on the definition coined by^40^: “The main feature of a bottleneck is that its downstream is in free flow and its upstream is jammed”. Thus, our method is based on the idea that if a bottleneck causes its upstream to be congested, the bottleneck must have been congested prior to it. Hence, for the definition of a bottleneck, time is as important as space.

## Results: Spatio-temporal dynamics of traffic bottlenecks—macro and meso-dynamics

We applied our method of identifying bottlenecks on three datasets of two urban areas (London, Tel Aviv, and the center of Tel Aviv—without the Ayalon Highway). We start by presenting the dynamics of the traffic bottlenecks, then demonstrate the importance of the global nature of the proposed method. We used data of near-real-time speeds to identify, at each time unit, the street segments that caused the traffic congestions (i.e. the traffic congestion’s bottleneck) and the streets that were affected by them later, as a result of the spillover process to the traffic congestion's upstream. Each street that becomes congested may affect other streets in its upstream (but not in its downstream), i.e. each road segment that acts as a bottleneck is linked to other congested streets that lead to it. This process creates a tree-shaped structure, where the bottleneck is regarded as its trunk.

To follow the spatio-temporal dynamics of the system, we combine all the different traffic congestions (represented as Jam Tree (JT)) with the same street acting as their trunk (source) throughout the entire examined week and refer to them as Repetitive Jam Trees (RJTs). The cumulative cost caused by an RJTs represents the sum of the cost of all the JTs they contain at a specific time window (e.g., day or week): ${TotalCost}_{RJT}=\sum {TotalCost}_{JT}$. For elaboration on the method and the way we calculate these quantities see Section methods and materials.

To address the dynamics of bottlenecks we present the analysis through two forms of order: scaling characteristics versus local meso dynamics. We found, that while the data correspond to scaling laws in a macro resolution^41,42^ when zooming into the spatio-temporal behavior of the bottlenecks and their trees, they vary both in their location and in the time they occur.

### Macro-stability

Previous work found universal laws in urban traffic congestion^42–48^. Some studies even identified a high degree of regularity in the measured speed of the street segments^41,42^. Others focused on the time evolution of urban congestion^45,47^, but not through the analysis of bottlenecks. At large scales, traffic dynamics and congestions have been found predictable^41,42^ and their weights follow power-law distributions^45,47^. With this in mind, we analyzed the bottlenecks' dynamic at the macro-scale to find if they present such regularities as well. We analyzed three temporal scales: the largest scale is a work week (that includes all the examined working days), the intermediate scale is a 24-h day, and the microscale is the different hours of the day.

#### Large scale—a 5-day work-week

**Figure 1:**
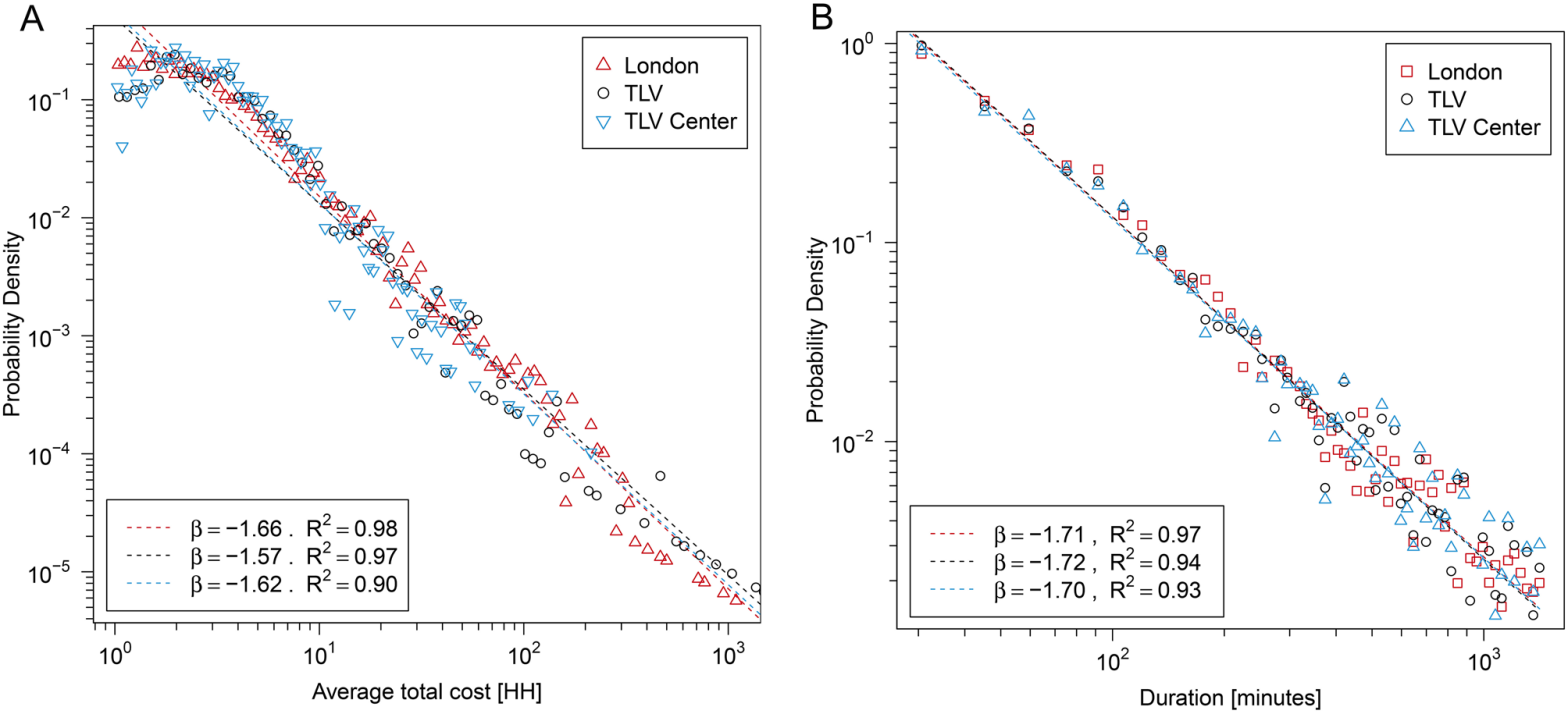
Analysis of the PDFs of the RJTs in London, Tel Aviv, and Tel Aviv Center (based on the data of all 5 days). (**A**) PDF of average cost, ${\text{Ave}}.{\text{Cost}}_{{{\text{RJT}}}} = \frac{{{\text{TotalCost}}_{{{\text{RJT}}}} { }}}{{\text{N}}}$, where N represents the number of JTs in the SJT; (**B**) PDF of duration (in minutes).

 We explored the behavior of the traffic congestions of the different datasets throughout the entire work-week and examined several attributes of the systems: the duration of the traffic congestions, their size (in terms of the number of road segments), and their cost (in VH units). Figure 1 shows that the distributions present similar behavior for all three datasets. For all datasets, the Probability density functions (PDFs) present, well approximated, power-law distributions. This implies that despite the different infrastructure and transportation facilities in these two cities, there may be common characteristics in London and Tel Aviv in terms of their traffic macro-dynamics. While these similarities concern the scaling of probabilities of having a bottleneck of a given size, they do not provide an answer to the question: “do the same bottlenecks repeat in time and space?” To try and answer this question, we analyzed the intermediate and short time scales as presented next.

#### Intermediate scale—Days

 We examined the PDF of $Max.{Cost(t)}_{RJT}$ for each dataset separately in each of the examined days (Fig. S1A–C) and found similar results. The data of London and Tel Aviv (including the Ayalon Highway) present a better fit to a power-law function than the data of Tel Aviv center (without the Ayalon highway). This may be, due to the fact, that the London dataset includes main roads (such as A501), which might have a similar effect on traffic dynamics as the Ayalon Highway. It is worth noting the resemblance between different days on each dataset.

#### Short scale—hours

 Lastly, we examined the dynamics of the RJTs in the different datasets and studied their behavior at different hours, days, and cities. By constraining the ${TotalCost}_{RJT}$ Eq. (4) to different time spans, we were able to evaluate their cost at different hours. For that, we sum the right-hand side of Eq. (4) between t_1_ and t_2_, where t_1_ and t_2_ correspond to the earlier and later times that define the examined time window. All three datasets present PDFs that correspond to power-law distributions with exponents between 1.8 and 2.4, where the morning rush-hours present slightly higher values than the afternoon rush-hours in all these cases (Fig. S1D-F). While the Ayalon Highway introduces costs that reach 1,000 VH per hour in both morning and afternoon rush-hours, in London the maximal cost per hour reaches 100 VH in the morning rush-hours, and in Tel Aviv Center, these values are even lower and reach 40VH per hour in the morning rush-hours.

We found that the characteristics of the macro-stability indeed align with previous findings, which may be explained by the fact that while different cities have different physical constraints, historical development, and socio-economic processes, urban road networks were developed based on similar principles, i.e., similar parameters of demand (urban travel) and supply (road infrastructure)^4^.

The results of the analysis of all these three temporal scales (large, intermediate, and short) show that the probability of having traffic congestion of a given cost is scale-free for all cities and in all time spans. Such PDFs can be useful for forecasting the existence of costly traffic congestion s (above a certain threshold) and the volume of their costs at different time scales. However, the values of these congestions (in VH units) and the exponents that govern their decrease with size, depend on spatio-temporal features and represent the different attributes of the different areas. These attributes can relate to the morphology of the street network^45,47^ or other factors such as different types of transportation methods available in each location, working hours norms, etc. Furthermore, while the distributions remain similar on different days in the same city, it is important to study, as we wish here, whether and how much the roads involved in the jam trees remain the same on different days. With this question in mind, we analyzed the meso-dynamics of the traffic networks.

### Meso-dynamics

While the bottlenecks cost appears at all the temporal scales in different time windows and the traffic load seems regular both in space and time, when zooming into the meso-dynamics of the traffic congestions, we unveil local characteristics that reflect shifts in the location of bottlenecks over time. These findings reinforce the need to develop a new framework for urban transportation, that is based on big data.

**Figure 2:**
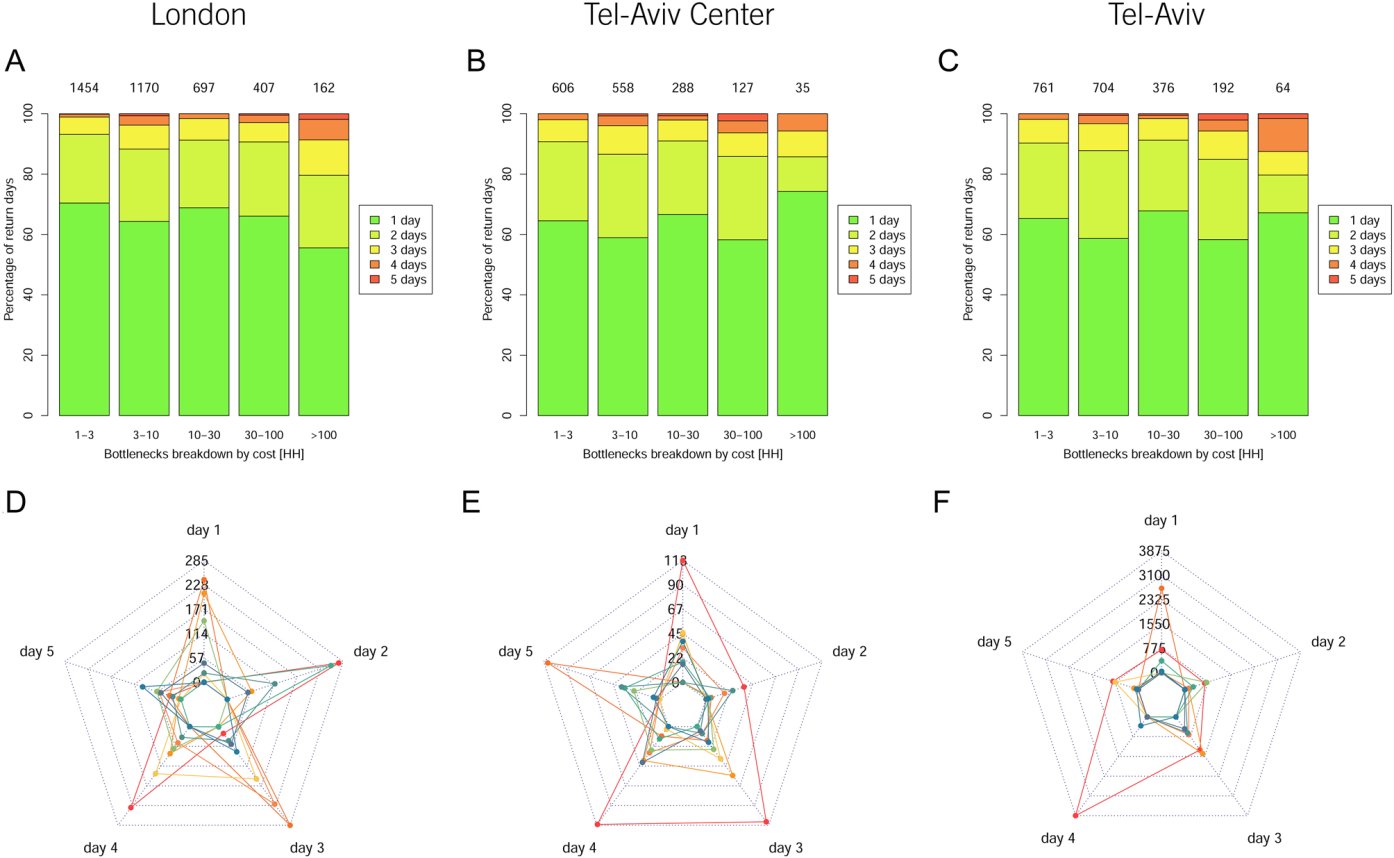
Top: the repetition of RJTs in (**A**) London (**B**) Tel Aviv Center (**C**) Tel Aviv. The X-axis represents the ${\mathrm{TotalCost}}_{\mathrm{RJT}}$ of the bottlenecks and the Y-axis represents the percentage of the bottlenecks with different ${\mathrm{TotalCost}}_{\mathrm{RJT}}$ repeated during the measured week. Bottom: the changes in the average ${\mathbf{T}\mathbf{o}\mathbf{t}\mathbf{a}\mathbf{l}\mathbf{C}\mathbf{o}\mathbf{s}\mathbf{t}(\mathbf{t})}_{\mathbf{R}\mathbf{J}\mathbf{T}}$ (Eq. 4) per hour (in VH units) for the 10 heaviest bottlenecks in (**D**) London (**E**) Tel Aviv Center (**F**) Tel Aviv in 5 days, during $\Delta \mathrm{t}$ = 4 h from t1 = 5 pm to t2 = 9 pm. These values have been obtained from Eq. 3 by summing the right-hand side between t1 and t2.

We analyzed the repetition of bottlenecks on different days and found that most of the bottlenecks are irregular and the same bottlenecks usually do not repeat daily (Fig. 2A–C). In all three datasets, close to 60% of all bottlenecks appear only in one day of the week. About 20% appear in two days and less than 10% of the bottlenecks with the same level of cost, appear in three days. Even when ignoring their cost levels (see Fig. S2), the number of bottlenecks that appear once or twice exceeds 60%. Thus, we see that most heavy traffic congestions do not repeat daily. Although in all three datasets the bottlenecks with the heaviest cost appear slightly more frequent (the percentage of bottlenecks with cost higher than 100 VH increases for bottlenecks that repeated in 4 or 5 of the days), there exist also many heavy bottlenecks that occurred only once or twice during the examined week. Thus, most of the bottlenecks are not predictable. These results also hold when assuming different thresholds for the bottlenecks’ ${TotalCost}_{RJT}$ (see Fig. S2). We also compared the above results to the analysis of the bottlenecks’ duration (in terms of hours) and found a similar behavior. i.e., bottlenecks that lasted longer tend to repeat slightly more frequently than the shorter ones (see Fig. S2). In other words, the heaviest bottlenecks (in terms of their duration and cost in VH units) are slightly more predictable than the short and less costly ones that occur on different days and locations. Nevertheless, as seen in Fig. 2, the number of bottlenecks that repeated 4–5 days is only 10–15% of all bottlenecks, whereas the heavy-loaded ones occupy less than 10% of them. These results are supported by the correlations between the occurrence of bottlenecks in space in time which show similar results (see Figs. S3 and S4 for elaboration).

We examined the variation of the ${TotalCost(t)}_{RJT}$ of the heaviest repeated bottlenecks on different days and at different hours and found that the hourly cost of these bottlenecks in all three datasets changes significantly on different days and hours in both size and rank (see Fig. 2D–F). These variations are more moderate in London, where the top five heaviest bottlenecks remain at the highest ranks (even though their cost significantly changes). In Tel Aviv, the situation is similar in terms of ranks, but the costs vary more significantly. This may be explained by the impact of the Ayalon Highway on the system, as explained earlier. When excluding the Ayalon Highway, Tel Aviv Center presents instability in terms of its bottlenecks' ranks and costs. This holds for all the three examined time spans (see Fig S5).

We also analyzed the cost caused by all JTs for different resolutions of measurement units T (see Materials and Methods section for elaboration). We found, for all 3 datasets, that when T increases (i.e. the resolution of the examination decreases) both the sum of the cost of all JTs and their variance decrease (see Fig. S6). This indicates that when increasing the time intervals between measurements, the data becomes flattened and important information (in this case the cost caused by traffic congestion) is lost. This strengthens the importance of following traffic dynamics in fine time scales.

**Figure 3:**
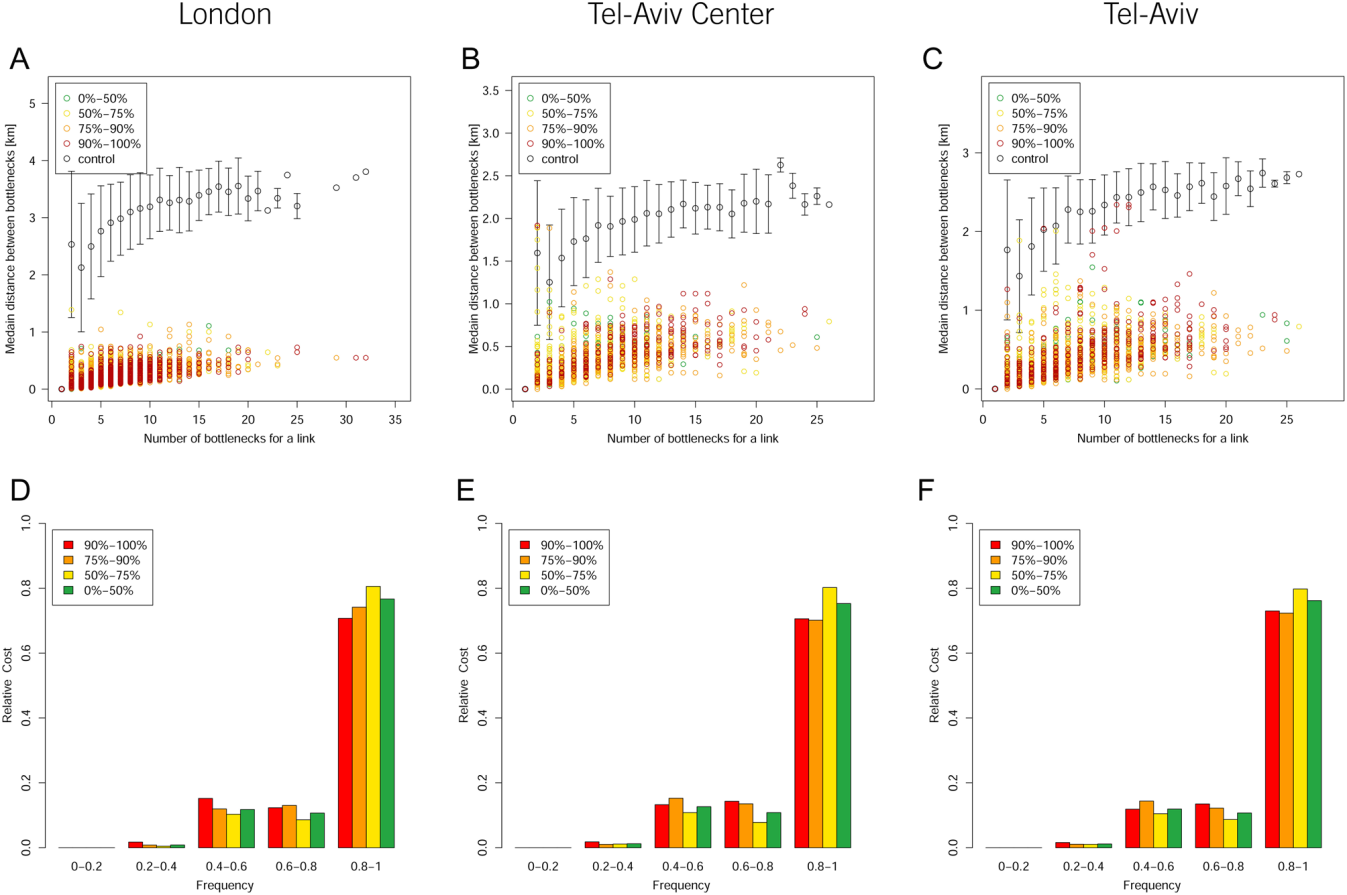
Top: the streets that were included in any of the JT during the examined week. The X-axis represents the number of different bottlenecks each street was connected to, the Y-axis represents the median distance between these bottlenecks, and the colors represent the relative percentage ${\mathrm{TotalCost}}_{\mathrm{RJT}}$ (Eq. 4) of each street, in comparison to the other streets in the dataset. The control group is calculated by allocating a number of random bottlenecks to each street as the number of different bottlenecks it was connected to. (**A**) London (**B**) Tel Aviv Center (**C**) Tel Aviv. Bottom: the distribution of the frequency of the relative ${\mathrm{CumulativeCost}(\mathrm{t})}_{\mathrm{JT}}$(Eq. 3), i.e., for each tree, the value of the trunk with the highest ${\mathrm{CumulativeCost}(\mathrm{t})}_{\mathrm{JT}}$, divided by the sum of the ${\mathrm{CumulativeCost}(\mathrm{t})}_{\mathrm{JT}}$ of all the branches that acted as trunks for this tree are presented for (**D**) London (**E**) Tel Aviv Center (**F**) Tel Aviv. The colors represent the level of costs of the JTs.

Next, we examined the dynamics of the bottlenecks that affected the jammed trees. For that, we analyzed (for each street that was part of any of the traffic congestions during our examination time window) the number of different bottlenecks it was connected to. Figure 3A–C shows for each of these streets (in all three datasets), the number of different bottlenecks they were connected to during the 5 days (x-axis), the median distance between these bottlenecks (y-axis), and their relative ${TotalCost}_{RJT}$ (colored symbols). These results show that congested streets are connected to a different number of bottlenecks (ranging between 1 and 22) regardless of their cost. However, the bottlenecks in London and Tel Aviv Center are located relatively in proximity to each other (their median distance is less than 1 KM) while the bottlenecks in Tel Aviv are spread over a wider area (up to 2.4 KM), which can be explained by the length of the Ayalon Highway (see above). This means, that while the traffic congestion can be associated with a specific area in the city, and even with some specific streets^38,40^, the location of the bottlenecks that causes the congestion changes constantly on different days and hours. This result suggests that constant, pre-defined solutions for traffic reduction cannot be the only solution to manage urban traffic congestion and strengthen the necessity of near real time analysis based on big data in order to improve urban traffic flow. Based on our proposed method, when a trunk is dissolved, the tree can continue to exist with another branch acting as its trunk. To further validate our results, we follow the distribution of the $Max.{Cost(t)}_{RJT}$ (maximal measured costs for Eq. 3) of each tree between all the branches that acted (at any time) as its trunk. Figure 3 (D-F) shows that in more than 80% of the cases where a tree has more than one trunk during its entire duration, there is a single dominant trunk that holds more than 80% of the tree ${CumulativeCost(t)}_{JT}$ (Eq. 3). In other words, in most cases, there is only a single trunk that is responsible for the JT. This analysis denotes the trunks as having a unique role in the evolution of traffic congestions, and thus emphasizes the need to address causality between the trunks and their JTs. We also calculated the same frequencies for the duration of the different trunks and found similar results (see Fig. S7).

### The importance of a global perspective of urban traffic bottlenecks

**Figure 4:**
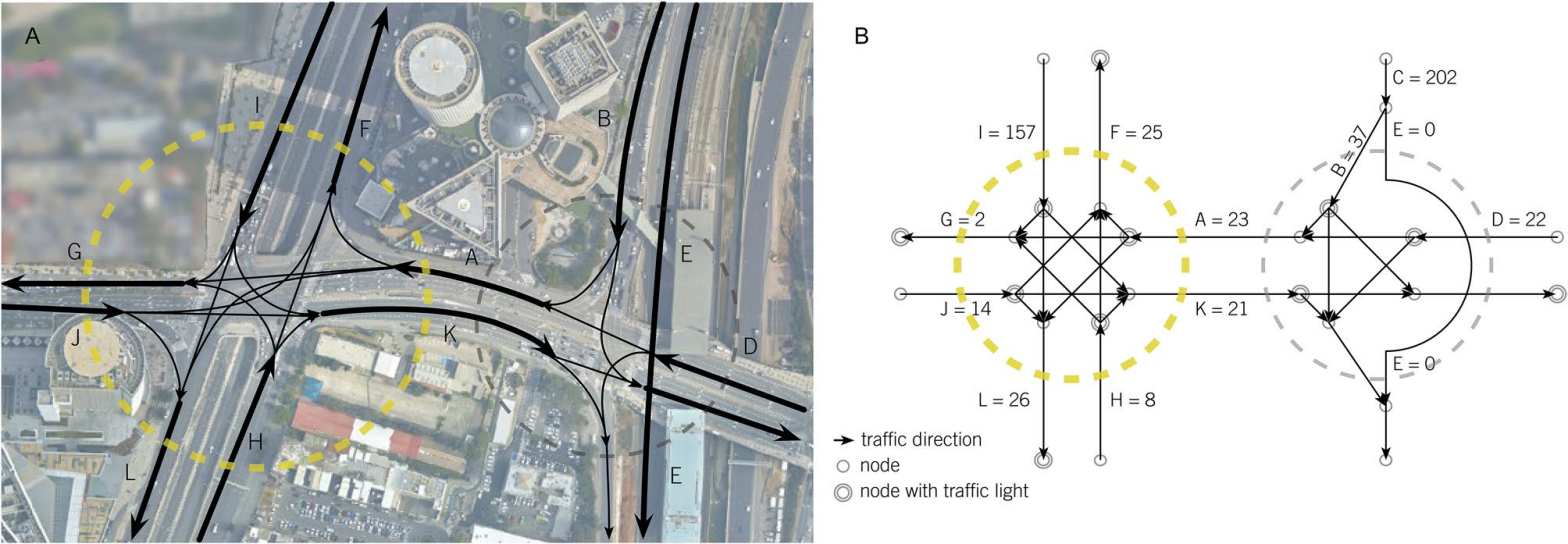
HaShalom Interchange on March 21st, 2018 at 8:45 am. (**A**) Aerial photo of the roads; (**B**) topological representation of the roads network, where the nodes represent junctions (with or without traffic lights), the links represent the road segments, and the weight of the links represents their ${\mathrm{CumulativeCost}(\mathrm{t})}_{\mathrm{JT}}$ (in VH units) from the time they became congested until 8:45 am. The areal photo was obtained from the Tel Aviv municipality GIS website: (https://gisn.tel-aviv.gov.il/iview2js4/index.aspx).

We present HaShalom Interchange as an example of the significance of considering the entire road network simultaneously and not analyzing traffic congestion at the local level. i.e., a single or a few adjacent junctions (see Fig. 4). This area connects several main roads as shown in Fig. 4. In our analysis, we focus on Ayalon South Highway (B, C, and E), Namir North (F), Kaplan West (G), Giv’at HaTahmoshet West (A), and HaSHalom West (D).

The analysis of the traffic congestion in this area is based on the real data, collected on March 21st, 2018, during the morning rush hours. When looking at the traffic congestion at 8:45 all the street segments (with the exception of E—Ayalon South) are congested. When analyzing the traffic congestion in this area locally, one can assume that road segment G (Kaplan West) may be the reason for the congestion that includes A, B, C, D, and I, as these roads connect to G. However, when analyzing the evolution of the JTs we found that G became congested only at 8:00, while A and D were the first road segments to become congested at 6:45, followed by B, and C (both at 7:15). Road segment I was naturally not affected by A, but it also became congested at 6:45 (an hour and 15 min before G). Further investigation reveals that A was not congested due to the congestions in F or L as well, as these roads became congested only at 7:45 an hour and 15 min after A became congested (see Fig. S8).

When converting these data into ${CumulativeCost(t)}_{JT}$ (in VH units, Fig. S8), from the time each road became congested until 8:45 am, we found the cost of G was 2VH, while the costs of A and I were 23 VH and 157 VH, respectively. The costs of F and L were 25 VH and 26 VH, respectively. Looking at these costs at the time of the examination, one might assume it is more important to address the congestions in I, however, the cost of the JT with A acting as its bottleneck (i.e. A, B, C, and D) was much higher, that is 284 VH, suggesting it is more important to address the congestion in A first. In fact, B and C are two lanes of the Ayalon Highway that lead to the HaShalom interchange. Thus, it also makes sense that congestion that affects this major highway causes larger damage than local urban congestion and should be addressed with higher priority.

The above example demonstrates that by solving the traffic congestion based on the analysis of individual junctions, one can miss the real bottlenecks that have the most significant effect on the overall road network. Naturally, the ability to prioritize the impact of different JTs in the city is not limited only to JTs that reach the same junction but can account for all JTs located in different areas of the city as well.

## Conclusions

We showed that although some universal power-laws distributions that appear daily, govern the macroscopic spatio-temporal behavior of sizes of traffic congestions, there are also unique behaviors that indicate that local attributes affect traffic dynamics as the same traffic bottlenecks usually do not reappear on different days. In other words, the macro-stability, presented by the scaling characteristics of the traffic bottlenecks that represent the seeming regularity of traffic load both in time and space, overshadows the existence of rich meso-dynamics, where the bottlenecks that create these JT loads, change significantly their location in time and space. This means that in order to manage traffic congestions in different locations and at different times and determine priorities of which ones should be addressed first, there is a need to implement unique solutions that track traffic and evaluate the relative effect of each bottleneck in real-time on the entire road network.

Urban traffic, like any other complex network, is composed of many elements and their interactions (e.g. street connectivity, land uses, public transportation, traffic light control system, etc.). Due to the numerous elements that affect urban traffic, it is almost impossible to predict its behavior. Thus, any intervention in a traffic light control system may lead to unexpected effects in other parts of the city, i.e. solving congestion in one location may divert the congestion to another place. This is one of the reasons for an urgent need of developing new, dynamic, real-time solutions that are based on big data that is retrieved, analyzed, and implemented in real systems in real-time (or near real-time). Such systems will keep adapting themselves based on the dynamics of the system, providing better solutions for improving urban traffic congestion. Such a dynamic optimization process is complicated and yet to be developed. This is due to the circular causality between the traffic lights and the actual traffic. However, in the era of the big-data revolution, it is only reasonable to assume that despite the computational challenges, such solutions could be developed. The proposed methodology here is a step toward such a goal, as it identifies simultaneously all traffic bottlenecks as soon as they emerge; it allows to accurately follow their propagation in near real-time even while intervening in the system, for example, by using an adaptive traffic light control system. Thus, our method can extend existing systems where big data are used to identify traffic congestions. Our methodology can assist in identifying and prioritizing the bottlenecks based on their cost (e.g., in human-hours units). This might require more accurate and detailed datasets, however, in the era of smartification of the cities, that can be obtained by IoT (Internet of Things) technologies (i.e., sensors) or ICT (Information and Communication Technologies, i.e., real-time navigation apps). By using such data, our framework could be used to develop new planning tools that allow increasing road supply by means of prioritizing the improvement of specific bottlenecks over the others. This can be done based on the bottlenecks' cost (as shown in this work) or based on other considerations such as evacuation during extreme events, helping emergency vehicles reach their destination faster, etc. Additionally, such a system can help reduce the demand by means of a dynamic road-pricing tool that is based on the relative cost each bottleneck causes. As this method is based on real data, it will be able to constantly feedback itself and better control and mitigate traffic congestion. Implementation of this method may be a part of real-life systems, leading to a breakthrough in dealing with urban traffic around the world.

## Materials and Methods

**Figure 5:**
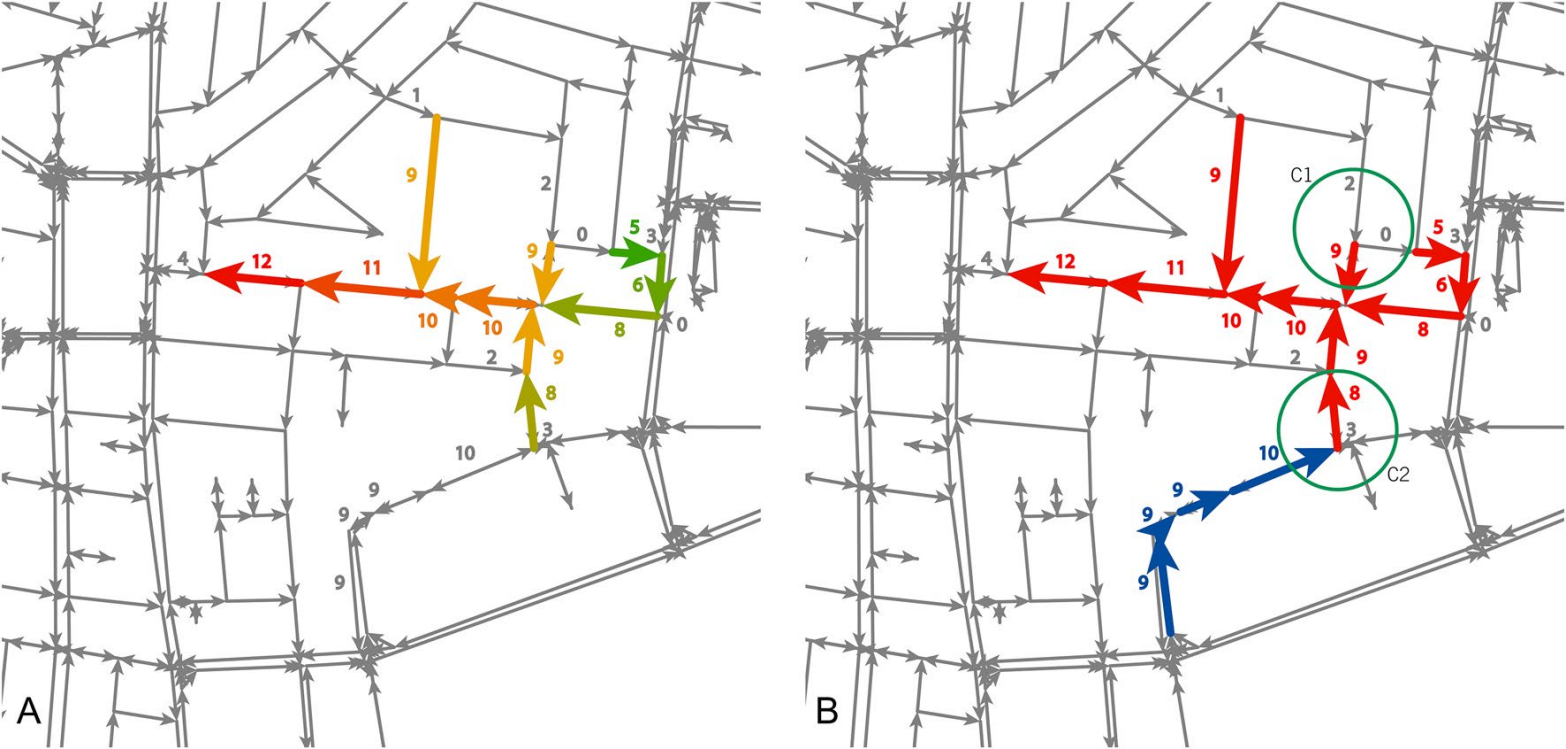
Clusters of JTs. The numbers represent the number of measurements each street segment was congested. (**A**) All the colored streets are part of one JT where the red street represents its trunk: its duration (12 successive measurements that represent 3 h) is the longest, which indicates it was the first street with traffic load in this JT. (**B**) Two JTs (represented by red and blue colors). The red JT does not include the street that has been loaded for 2 measurements, as the time gap between this street and its adjacent one is larger than the pre-defined threshold $\uptheta$ (see the upper green circle). The blue JT cannot be considered as part of the red JT, as the duration of its trunk is longer than that of its adjacent street in the red JT (see the lower green circle). When a bottleneck is released but the JT that follows it remains congested, the next street segment with the longest duration becomes the new trunk of the JT and carries the cost of the remaining branches of the JT.

To identify traffic bottlenecks, we converted datasets of urban areas to dynamic, directed traffic networks where each node represents a junction, and each link represents a street segment between two junctions. The direction of the links represents the allowed traffic on that street segment, and the weight of the link at time segment $t$, $W(t)$ represents the temporal traffic relative speed, i.e. the ratio between the temporal speed and the speed at its maximal flow. We defined a street segment as currently congested if ($W(t))<1$ (see Fig. S9 for elaboration). Next, we construct for a given time $t$ a new dynamic weighted network, where $W^{\prime}\left( t \right)$ is the cumulative continuous time each link has been considered as congested at $t$ (see Fig. 5) and used the following process to create tree-shaped clusters of congested links:

1. At each time $t$, we identify the links with the highest weight $W^{\prime}$ (i.e. that have been congested the longest time) and define them as potential trunks of a jam-tree (JT). Next, we identify the branches of the JT by adding links or other trunks, connected to each trunk, with $W^{\prime} \le W^{\prime}_{trunk}$. By doing so, we identify links that became congested no more than a predefined parameter $\theta ,$ in this case—defined as 2 measurement units, after the trunk. The value of $\theta$ is only used to limit the connections of new branches to a JT; in other words, it reflects the maximal duration that a congested street segment is considered as the cause for the congestion in its upstream. High values of $\theta$ allow a street segment to connect to its downstream longer times after its downstream became congested. This leads to larger JTs on one hand but reduces the probability of causality on the other. In other words, in our analysis, if a street segment became congested no more than 30 min after its trunk we can assume that the traffic load in these links resulted from the trunk of the JT. To test this assumption, we compared the result of the analyses of the real data to those of a control random model. The results of this comparison present a qualitative difference, which strengthens our assumption of causality (see Fig. S10 for elaboration). By using this definition, we consider the street segment that acts as the trunk as a bottleneck of the JT. Note, that we chose $\theta =2$ as our datasets had 15 min time-intervals and thus, our analysis considered the macro-dynamics of urban traffic. For other datasets with higher resolution of shorter time intervals, different values of $\theta$ can be used.
2. We continue assigning connected links to these JTs in the same iterative process until no more connected links (roads) with $W^{\prime} \le \theta$ for the last added branches are found.
3. We start again at stage 1, but now we look for the link with the highest weight $W^{\prime}$, that has not been assigned to an existing (JT).
4. We continue this process until there are no more congested links that are not assigned to any JTs.

The resulted clusters represent JTs and the time each of their links was loaded. Examples of JTs are shown in Fig. 5.

While some traffic congestions can last many hours, their economic cost might be marginal, if, for example, they occur in peripheral small streets. To assign prioritization for traffic congestions, we measure their cost in vehicle hours (VH). In order to calculate the cost at different times of the JTs and the links they include, we introduce the following formulas.

The cost of a link ${C}_{ij}(t)$ is calculated for every measurement unit − 15 min in this case, relative to its cost ${U}_{f}$, free-flow speed. This measurement unit demonstrates the meso-dynamics of urban traffic. Indeed, using shorter periods of time will allow following the micro-dynamics of urban traffic. This cost represents the time it takes to cross a road (link) in comparison to the time it takes to cross this road in a maximal flow (calculated for each link), multiplied by the number of drivers who crossed the endpoint of this link at a specific time:

1 $$C_{ij} \left( t \right) = dist_{ij} *\left( {\frac{1}{{u_{ij} \left( t \right)}} - \frac{1}{{u_{{q_{\max } ij}} }}} \right)*\frac{{q_{ij} \left( t \right)*l_{ij} }}{\frac{60}{T}}$$

here ${{\varvec{d}}{\varvec{i}}{\varvec{s}}{\varvec{t}}}_{{\varvec{i}}{\varvec{j}}}$ is the length of the link in km, ${{\varvec{q}}}_{{\varvec{i}}{\varvec{j}}}\left({\varvec{t}}\right)$ is the current flow on the link, ${{\varvec{u}}}_{{\varvec{i}}{\varvec{j}}}\left({\varvec{t}}\right)$ is the current speed on the link, ${{{\varvec{u}}}_{{\varvec{q}}{\varvec{o}}}}_{{\varvec{i}}{\varvec{j}}}$ is the speed when the flow is optimal, ${\varvec{T}}$ represents a measurement unit which corresponds to 15 min (in the present case) and ${{\varvec{l}}}_{{\varvec{i}}{\varvec{j}}}$ is the number of lanes in the link (i.e. the number of lanes in each street segment of the JT).

The momentary cost of a JT represents the sum of the costs (Eq. 2) of all the links that are included in it at a specific measured time:

2 $${\varvec{MomentaryCost}}\left( {\varvec{t}} \right)_{{{\varvec{JT}}}} = \user2{ }\mathop \sum \limits_{{{\varvec{b}}_{{{\varvec{ij}}}} }}^{{\varvec{n}}} \left( {{\varvec{C}}_{{{\varvec{ij}}}} \left( {\varvec{t}} \right)} \right)$$

And the cumulative cost of a JT is the cost of the JT from the moment it was created until the time (t) which is calculated as:

3 $$CumulativeCost\left( t \right)_{JT} = \mathop \sum \limits_{{b_{ij} }} \left( {\sum\nolimits_{{t_{I} \le t}}^{t} {C_{ij} \left( {t_{I} } \right)} } \right)$$

Here, ${b}_{ij}$ is a branch (i.e. link) in the JT and ${t}_{I}$ is the time each branch ${b}_{ij}$ was a part of the JT (in 15 min units).

**Figure 6:**
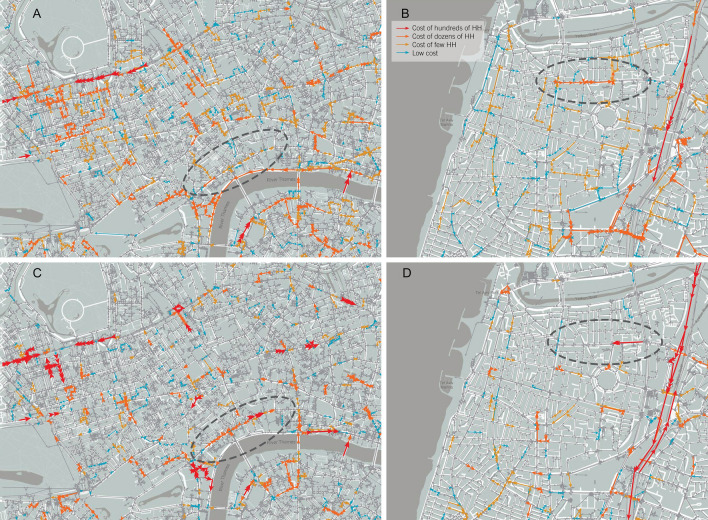
Graphical representation of the JTs: snapshots of JTs in (**A**, **B**) London center and (**C**, **D**) Tel Aviv and the ${\mathrm{CumulativeCost}(\mathrm{t})}_{\mathrm{JT}}$ of the entire congestion, associated to each bottleneck, during morning rush hours (up) and evening (bottom). While some traffic congestions appear in both morning and afternoon rush hours (e.g., Marylebone street in London, or the Ayalon Highway in Tel Aviv), Others are congested in only one of the rush hours. For example, Victoria Embankment road between Black friars Bridge and Waterloo Bridge (see black circle) is heavily congested only in the morning rush-hour snapshot; and Pinkas St. in Tel Aviv (see blue circle) is congested only in the evening rush hours snapshot). The maps were created using Snazzy Maps (https://snazzymaps.com/help), Rhino5 https://www.rhino3d.com/download/archive/rhino/5/latest/), and Grasshoper plugin (https://www.grasshopper3d.com/page/download-1).

Figure 6 demonstrates some examples for different ${{\varvec{C}}{\varvec{u}}{\varvec{m}}{\varvec{u}}{\varvec{l}}{\varvec{a}}{\varvec{t}}{\varvec{i}}{\varvec{v}}{\varvec{e}}{\varvec{C}}{\varvec{o}}{\varvec{s}}{\varvec{t}}({\varvec{t}})}_{{\varvec{J}}{\varvec{T}}}$, based on real data for London and Tel Aviv.

Lastly, to follow the spatio-temporal dynamics of the system, we combine all the different JTs that had the same street as their trunk throughout the entire examined week and refer to them as Repetitive Jam Trees (RJT). The cumulative cost of the RJTs represents the sum of all the JTs they contain at a specific time window (e.g., day or week):

4 $$TotalCost_{RJT} = \sum TotalCost_{JT}$$

Equations (2)–(4) allow calculating not only the cost of each JT from the moment it became congested until it was dissolved but also its dynamics and temporal costs at different times (see Fig. 6).

### Data

The results of our framework and analysis are demonstrated for three urban areas: London center, Tel Aviv center (including the Ayalon highway–the main road that crosses the city from North to South), and Tel Aviv Center (without the Ayalon highway). These cases were chosen as they represent cities of different scales (London center is 2.5 times larger than Tel Aviv center); different public transportation systems and different regulations that influence the driving behavior; and the exclusion of the Ayalon highway from the data of Tel Aviv also allows us to explore the effect of a local highway on the local transportation characteristics.

We collected from Google Directions API the speeds of 8857 road sections in London center and 2,950 road sections in Tel Aviv (2324 of which are of Tel Aviv Center), every 15 min over a week's time. The data for London center was collected between the dates 21-27/3/2018 and the data for Tel Aviv center has been collected between the dates 12-18/2/2017). We developed an algorithm that considers additional road segments (for which we did not have data) based on interpolating the data collected for their adjacent road segments and ended up with 18,050 road sections in London, 5425 road sections in Tel Aviv, and 3871 road sections in Tel Aviv Center. For each case, we analyzed the data of 5 working days only (Mon-Fri in London and Sun-Thursday in Tel Aviv). This is because the results indicate that the dynamics of these systems are significantly different during the weekends.

## Supplementary Information

Supplementary Information.

## Data Availability

For contractual reasons, we cannot make the empirical data from Google Direction available. However, all data from our analysis are available at GitHub: https://github.com/nimrodSerokTAU/bottlenecks-prioritization.

## Code availability

The code of our analysis is also available at GitHub: https://github.com/nimrodSerokTAU/bottlenecks-prioritization.
