# Supplementary material for: Identification, cost evaluation, and prioritization of urban traffic congestions and their origin
Source: Sci Rep. 2022 Jul 29;12:13026. doi: 10.1038/s41598-022-17404-8 (PMC9338062; doi:10.1038/s41598-022-17404-8)
Supplement: Supplementary file 1 — Supplementary Information. [file 41598_2022_17404_MOESM1_ESM.pdf]

Identification, cost evaluation and prioritization of urban traffic congestions and their origin – Supplementary Information

Nimrod Serok[1], Shlomo Havlin[2], Efrat Blumenfeld Lieberthal[1*]

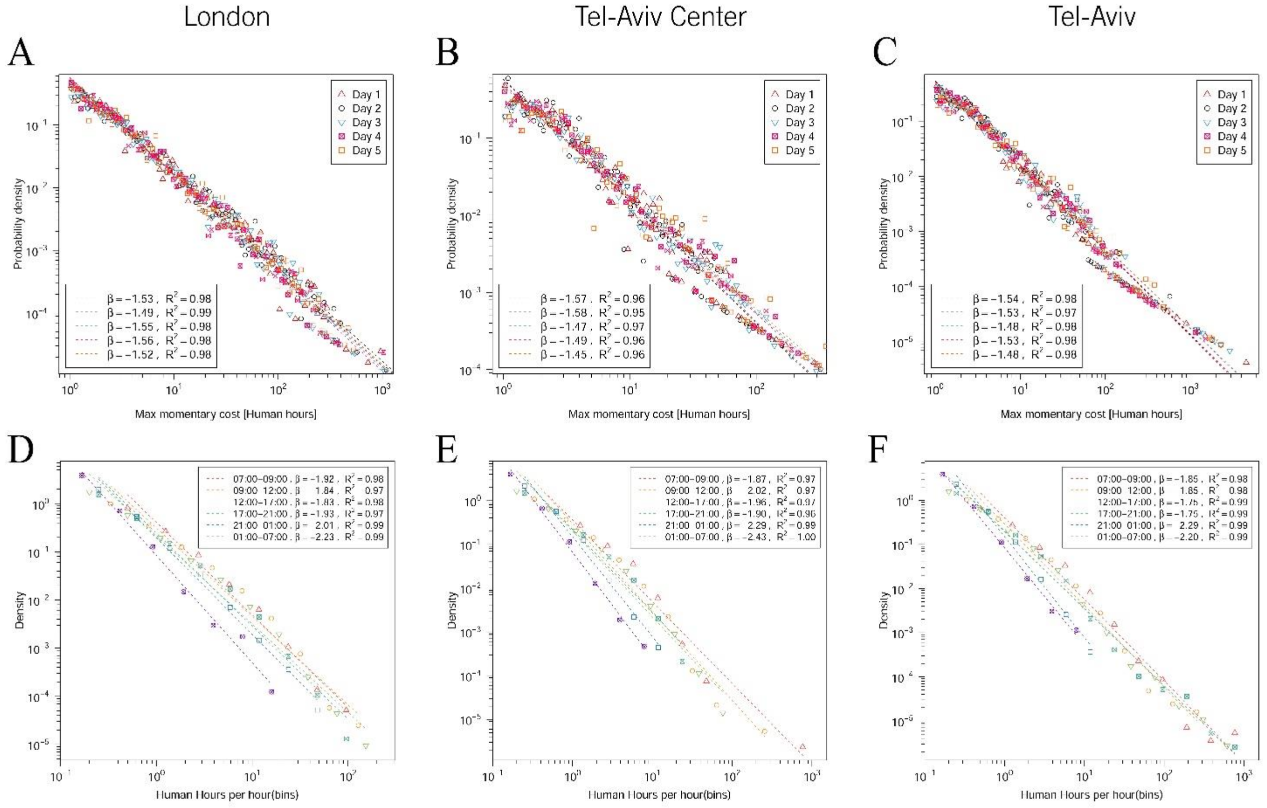

Figure S1: Top: The PDF of the $\mathbf{MomentaryCost(t)_{JT}} = \sum_{\mathbf{b_{ij}}}^{\mathbf{n}}\left(\mathbf{C_{ij}(t)}\right)$ **(eq. 4)** for **(A)** London **(B)** Tel Aviv Center, and **(C)** Tel Aviv. **Bottom: The PDF of** $\mathbf{TotalCost(t)_{RJT}}$ **(eq. 6) per hour** for **(D)** London **(E)** Tel Aviv Center **(F)** Tel Aviv in VH units.

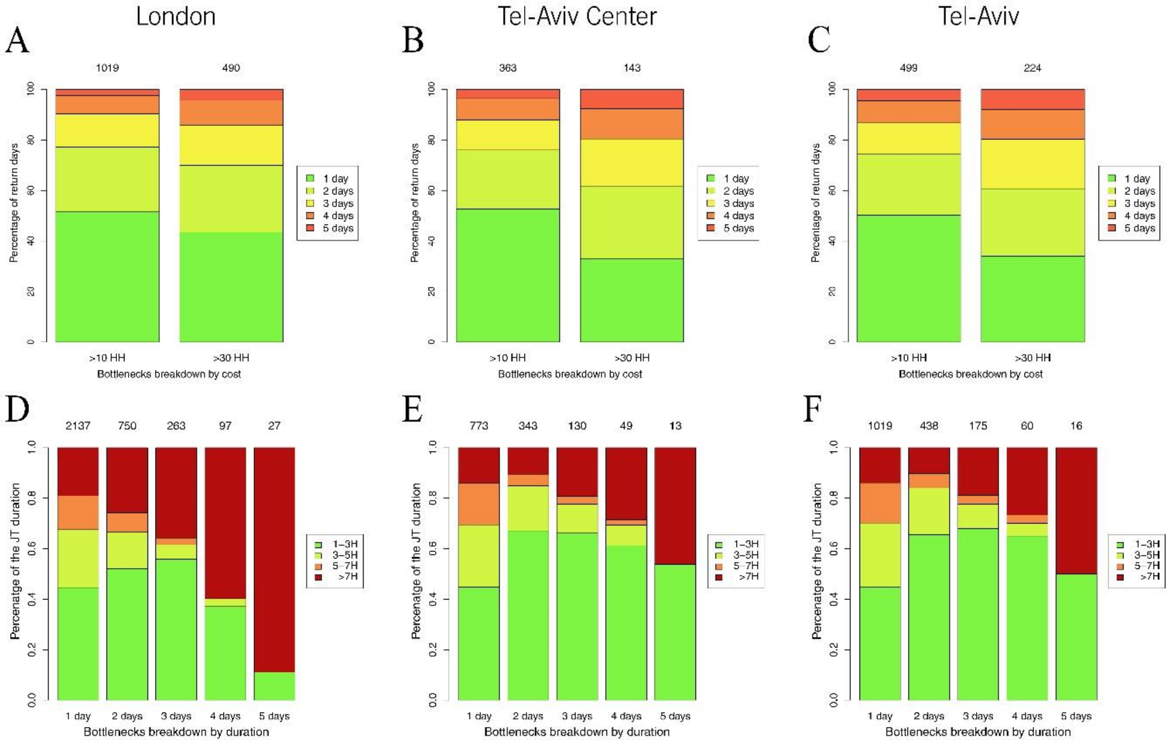

Figure S2: Top: The repetition of bottlenecks in different number of days for **(A)** London **(B)** Tel Aviv Center **(C)** Tel Aviv. The X-axis represents $\mathrm{TotalCost}_{\mathrm{RJT}}$ (eq. 6) and the Y-axis represents the percentage of bottlenecks that repeated once, twice, three, four, or five days over the examined week. More than 60 percent of all bottlenecks repeated only once or twice during the examined week. This applies to heavy traffic congestions as well as to lighter ones. The traffic bottlenecks that repeated in 4 or in 5 of the examined days represent only 10-20 percent of all traffic bottlenecks. However, in all three datasets we found that heavier bottlenecks tend to repeat slightly more frequently than lighter ones. **Bottom: The repetition of bottlenecks on a different number of days** for **(D)** London **(E)** Tel Aviv Center **(F)** Tel Aviv. The X-axis represents bottlenecks that repeated once, twice, three, four, or five days over the examined week. The Y-axis represents the distribution of the duration of these bottlenecks (in percentage). More than 80 percent of the bottlenecks occurred only once or twice. About half of the congestions that occurred only once lasted less than 3 hours, such traffic congestions compounded only 20 percent of the traffic congestions that repeated in the five sampled days in London. On the other hand, long traffic congestions (that lasted for 7 hours or more) occupied about 10-20 percent of the bottlenecks that occurred only once, but their percentage increased to 40 and 90 percent of the bottlenecks that repeated 5 days in Tel Aviv and London correspondingly. In other words, this analysis shows that longer traffic congestions tend to appear more frequently than shorter ones.

We calculated the cross correlations between the $\mathrm{TotalCost}_{\mathrm{RJT}}$ (eq. 6) of the RJTs for different days using both Pearson and Spearman correlations (figure S4). These correlations also show that the bottlenecks present better correlations for the heaviest traffic congestions (in terms of their cost in VH units) which indicates that these bottlenecks tend to appear more frequently than other. An exception for this behavior is Tel Aviv where the Pearson correlations of its heaviest bottlenecks increase inversely to their cost (figure S4B.). This can be explained by the dynamics and the congested nature of the Ayalon Highway. The Ayalon Highway is the main road that crosses Tel Aviv from North to South and connects the city to Haifa in the North and Jerusalem in the South East. Even though it is usually congested during rush-hours, the location of the bottlenecks that cause the traffic congestions changes on different days and hours, since there are many adjacent interchanges that connect this road to the center of Tel Aviv, and their congestion is irregular. The increase in the values of the correlations for the less costly bottlenecks corresponds to the fact that many traffic congestions occur only once which leads to higher probabilities of finding fluent traffic in most of the days for most of the streets. As Pearson correlation is highly affected by the large values of the sample, we add an analysis of the Pearson correlation for the tail of the distribution (see figure 5 C) . It reveals that indeed, the correlation of most of the bottlenecks between different days is low (reached -0.2). It corresponds to the fact that these bottlenecks do not repeat on different days. Only the most costly bottlenecks (top 5 percent), present the best correlations, i.e. they tend to appear on most of the days.

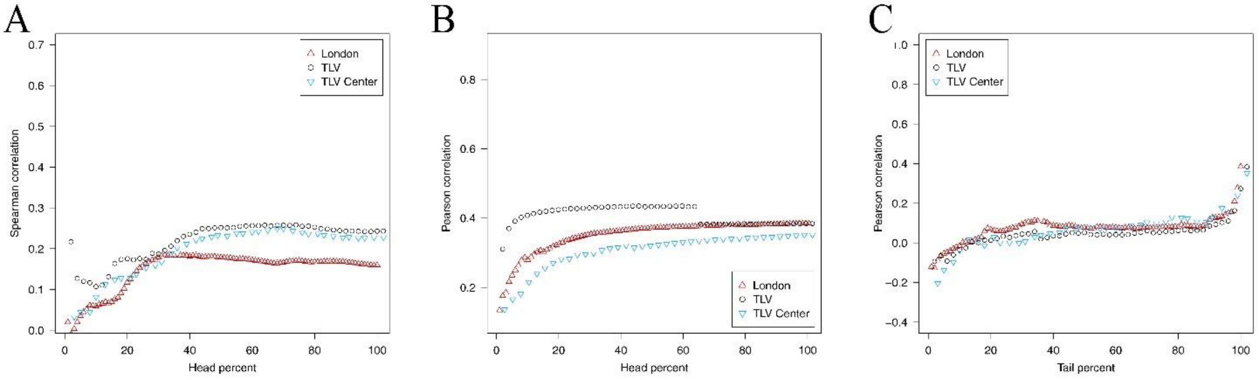

Figure S3: Correlations between the re-occurrence of traffic bottlenecks at different days of the week. (A) Spearman correlation **(B)** Pearson correlation. The X-axis represents the percentage of traffic bottlenecks included in the analysis (where the small values correspond to the heaviest bottlenecks in terms of their cost in VH units). **(C)** Pearson correlation where the X-axis represents the percentage of traffic bottlenecks included in the analysis (where the small values correspond to the least heavy bottlenecks in terms of their cost in VH units).

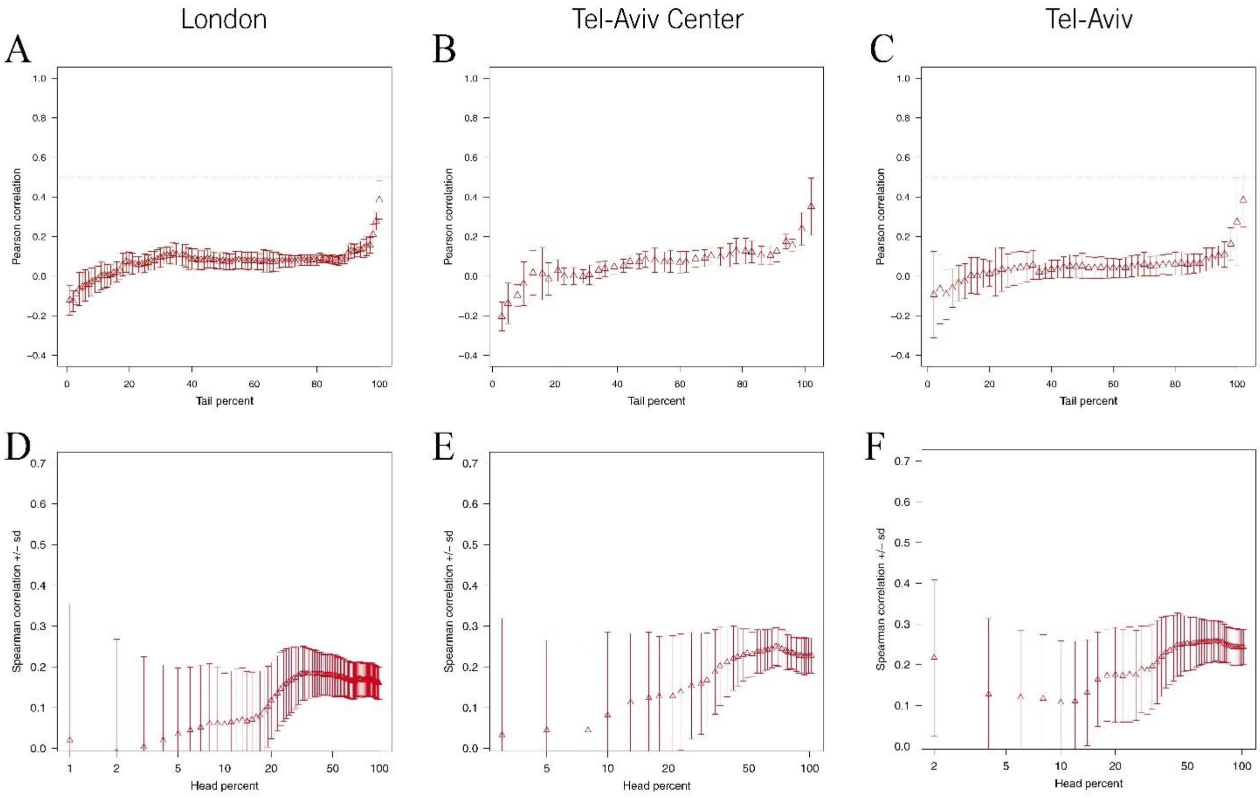

Figure S4: Top: Pearson correlations and the standard deviation of their values for **(A)** London **(B)** Tel Aviv Center **(C)** Tel Aviv. These correlations are based on the lower tail (head) of the data. This analysis shows that the largest errors occur for the highest values of the Tel Aviv and Tel Aviv Center data (i.e. the heaviest bottlenecks in terms of their cost). In London, however, the error is slightly smaller for all data, including the highest values that represent the correlations between the most costly bottlenecks. These results also support out previous observation – the heaviest traffic congestions in Tel Aviv are rooted in bottlenecks that shift in space and time. London, on the other hand, is characterized by a rather stable behavior of its traffic congestions and bottlenecks' spatio-temporal dynamics.

Bottom: Spearman Correlations and the standard deviation of their values for **(D)** London **(E)** Tel Aviv Center **(F)** Tel Aviv. X-axis presents the upper tail (head) of the data and is presented on a logarithmic scale. All datasets present a similar behavior as the error is larger for the heaviest bottlenecks and decreases for the less costly ones. As Spearman correlation is based on ranks rather than values (see Pearson correlations for values), this data shows that the heaviest bottlenecks are less stable than the less costly ones in term of their rank. In other words, this means that the most costly bottlenecks in the different days are placed in different streets. We compared these correlations to a random model, and found that the values of the real correlations are significantly higher than the other correlations, resulted by our random test. This

validates the accuracy of the correlation test and the resulted conclusions. Note, that for Tel Aviv Center we present the correlation between days 1,2,4, and 5 as day 3 occurred on February 14th which is Valentine day. In the dataset of Tel Aviv there are some random correlations that resemble the real ones. This might be a result of the heaviest bottlenecks on the Ayalon Highway that shift from one junction to another in different days.

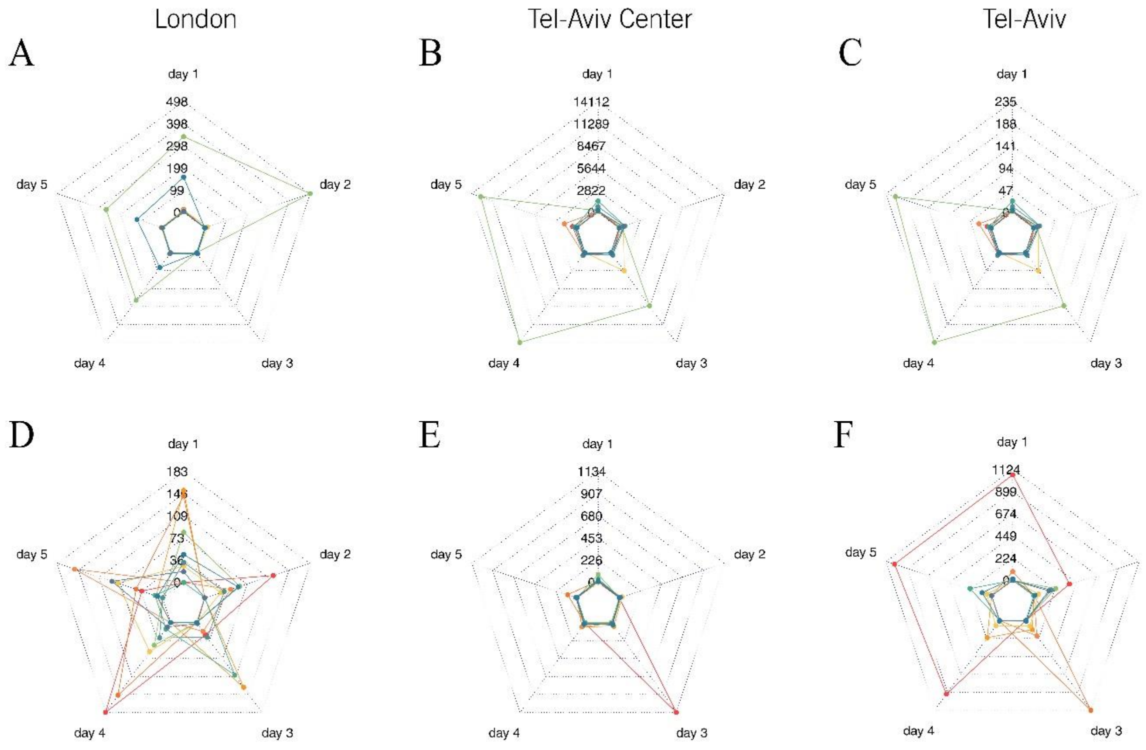

Figure S5: The change in the average $\mathbf{TotalCost(t)_{RJT}}$ (eq. 6) per hour (in VH units) for the 10 heaviest bottlenecks in **(A)** and **(D)** London **(B)** and **(E)** Tel Aviv Center **(C)** and **(F)** Tel Aviv, in 5 days: **(A), (B), (C)** $\Delta t=24$ hours where t_1=7am t_2=6:59am in the next day. **(C), (D), (F)** $\Delta t=2$ where t_1=7am t_2=9am.

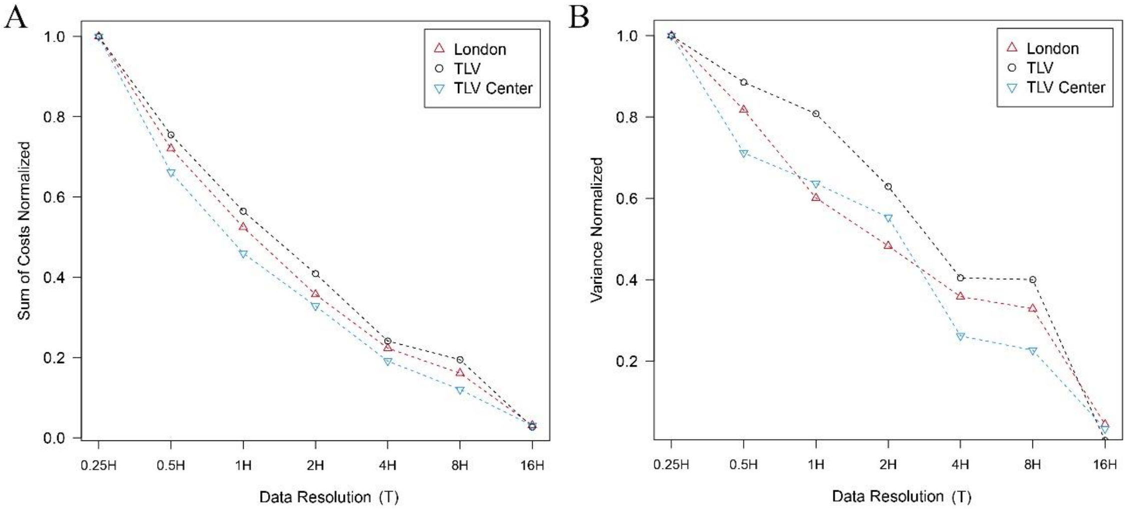

Figure S6: The effect of T on the sum of the costs of all JTs (A) and their variance (B) in London Tel Aviv, and Tel Aviv Center: When T (the measurement unit) increases the sum of costs, as well as their variance decreases. This indicates that for larger, less granulated measurements units, important data is lost, and the analysis yields a distorted picture about the cost of the congestion.

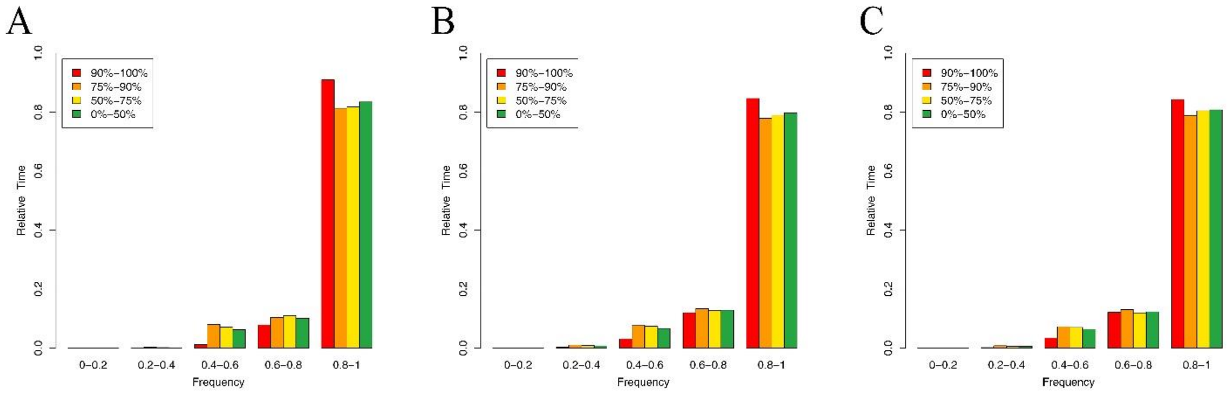

Figure S7: The distribution of the frequency of the relative duration of each of the branches that acted as trunks for a specific JT in **(A)** London **(B)** Tel Aviv Center **(C)** Tel Aviv. More than 80 percent of these branches acted as trunks for the largest percentage of time (80% or longer) which supports our claim that there is a unique, dominant trunk for each of the JTs.

	A	B	C	D	E	F	G	H	I	J	K	L
Number of lanes	4	4	5	5	4	4	2	6	5	4	4	3
Speed availability threshold (U_{jam}) [Km/h]	27	36	60	18	63	28	30	42	20	14	32	21
Free flow speed (U_f) [Km/h]	46	60	104	34	105	48	52	67	34	30	58	36
Segment length [m]	150	280	650	90	630	600	170	160	600	170	150	250
Speed at measurement [Km/h]												
06:30	28	49	88	23	90	27	52	37	21	29	42	22
06:45	22	43	82	19	82	31	39	33	15	29	42	17
07:00	22	42	72	11	73	29	39	42	19	29	36	34
07:15	19	35	54	12	61	29	39	45	16	7	29	21
07:30	15	34	32	12	73	29	39	35	16	6	29	22
07:45	17	22	28	10	75	19	31	45	9	6	24	18
08:00	17	20	19	12	63	23	26	56	16	29	17	12
08:15	17	20	22	10	48	25	26	35	10	29	15	12
08:30	23	20	23	9	73	25	26	28	8	7	18	10
08:45	17	21	26	12	73	25	26	35	6	5	19	8
Cost at measurement [HH]												
06:30	0.0	0.0	0.0	0.0	0.0	0.9	0.0	1.3	0.0	0.0	0.0	0.0
06:45	1.4	0.0	0.0	0.0	0.0	0.0	0.0	2.5	9.8	0.0	0.0	1.8
07:00	1.4	0.0	0.0	2.7	0.0	0.0	0.0	0.0	1.6	0.0	0.0	0.0
07:15	2.4	0.3	3.8	2.2	0.8	0.0	0.0	0.0	7.5	5.9	0.7	0.0
07:30	4.1	0.7	23.9	2.2	0.0	0.0	0.0	1.9	7.5	7.1	0.7	0.0
07:45	3.2	6.3	28.8	3.2	0.0	10.8	0.0	0.0	27.6	7.1	2.0	1.3
08:00	3.2	7.6	41.7	2.2	0.0	5.3	0.5	0.0	7.5	0.0	4.5	4.9
08:15	3.2	7.6	37.1	3.2	7.7	3.0	0.5	1.9	24.1	0.0	5.4	4.9
08:30	1.1	7.6	35.6	3.8	0.0	3.0	0.5	4.3	31.3	5.9	4.1	6.4
08:45	3.2	6.9	31.4	2.2	0.0	3.0	0.5	1.9	39.8	8.4	3.7	8.2
Cost by 08:45 [HH]	23	37	202	22	0	25	2	8	157	14	21	26

Figure S8: Real data for the Number of lanes, U_{jam}, U_f, Length, speeds, $\mathbf{MomentaryCost(t)_{JT}} = \sum_{b_{ij}}^{n}\left(C_{ij}(t)\right)$, and $\mathbf{CumulativeCost(t)_{JT}}$ for t=8:45 on which we based the example presented in figure 4.

To calculate $W(t)$, we need data of the speed at the maximal flow of each link. There are many approaches to address traffic stream and evaluate urban flow (see a comprehensive review in (1). We follow (1) who suggested a generalized car-following model that bridges microscopic and macroscopic models:

$$(1)\ U^{1-m} = U_f^{\ 1-m}\left[1 - \left(\frac{k}{k_j}\right)^{l-1}\right]$$

Here, U represents the speed, U_f represents the free-flow speed, k represents the density, and k_j represents traffic congestion density. For each street segment, we extracted U_f as the 95% percentile of the maximal measured speed. For each street and each measurement, we calculated k (based on eq. 2) and q $(q\ is\ the\ Flow = k * U)$. Using this formula allows to extract the maximal potential flow of each street segment (even without the empirical measurement of it) and its matching speed.

Figure S9 presents the CDF of the $W(t)$ of 8,857 road sections in London center, 2,950 road sections in Tel Aviv, and 2324 road sections in Tel Aviv Center for 96 measurements every day (every 15 minutes) during 5 days of a work-week.

In all the datasets, about 20% of the examined road segments measurements were congested during the examined week (W(t) < 1), while the congestions in London were more severe than the ones in Tel Aviv (about 10% of the road segments measurements in London presented $W(t)$<0.5, while only 5% of the road segments measurements in Tel Aviv presented values of $W(t) < 0.5$).

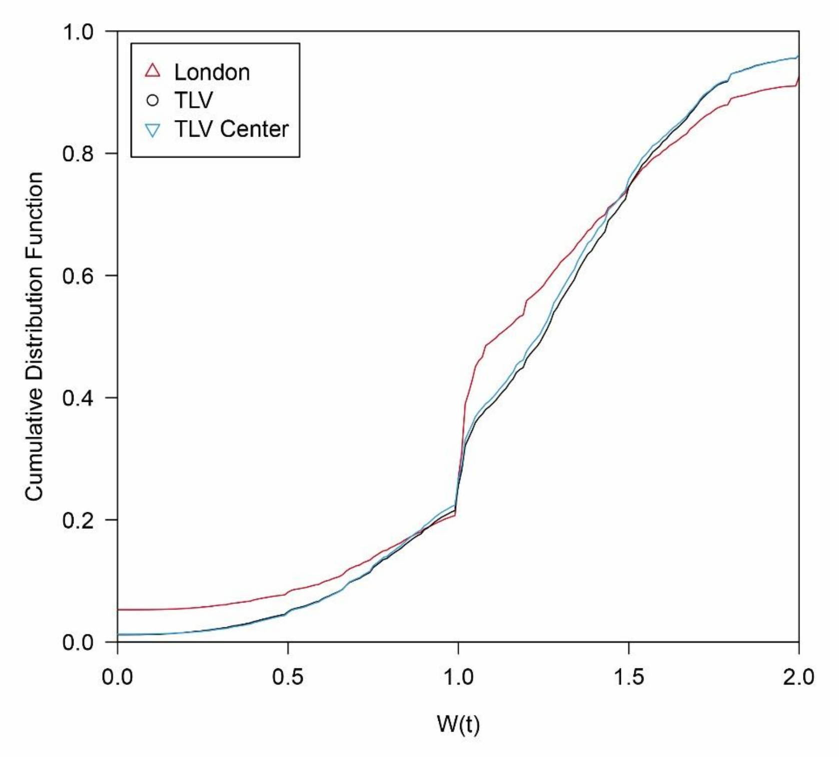

Figure S9: CDF of W(t) for London, Tel Aviv, and Tel Aviv Center.

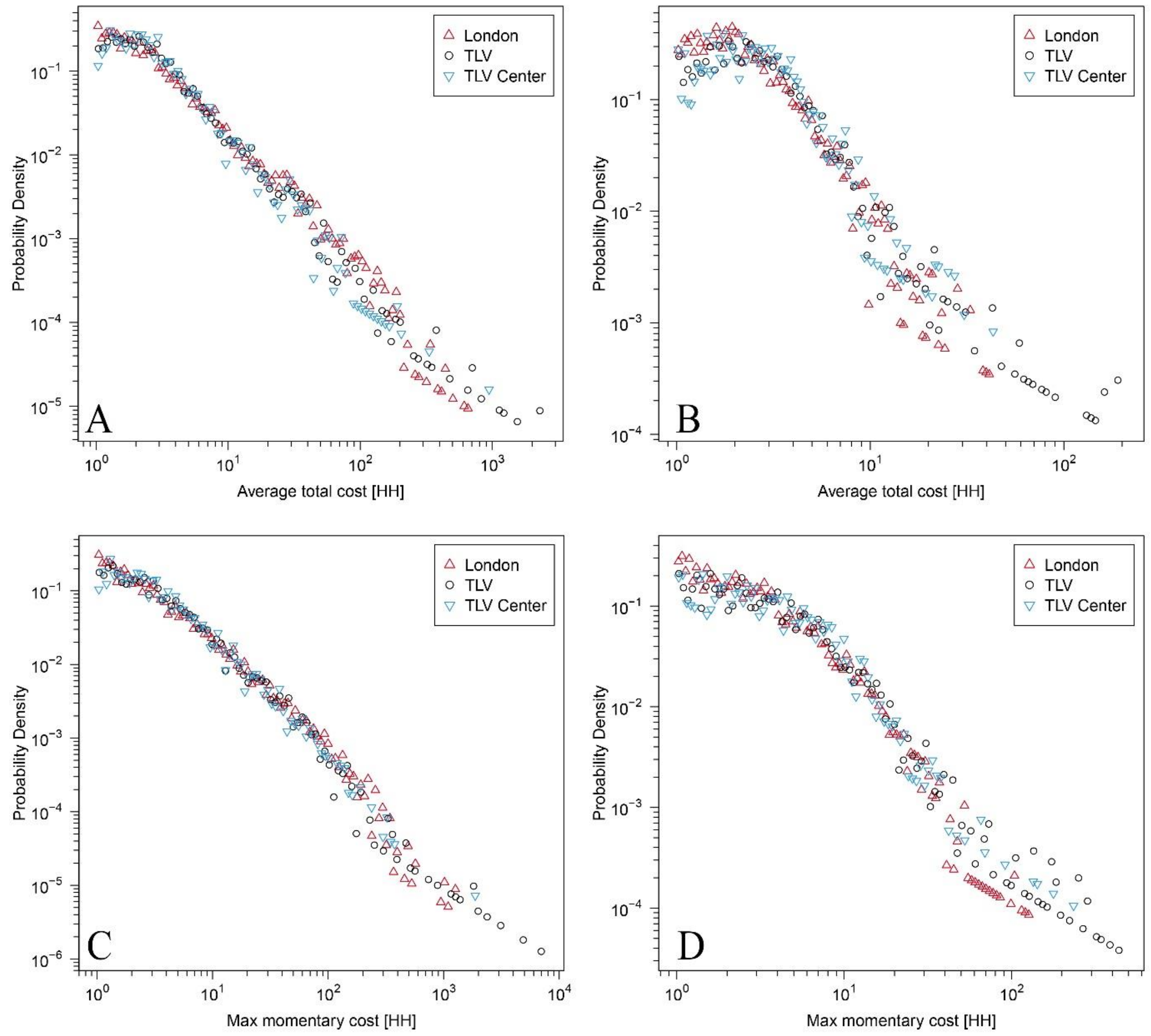

Figure S10: Comparison between the results of the real data and a random model for the PDFs of the RJTs in London, Tel Aviv, and Tel Aviv Center (based on the data of all 5 days): $\mathbf{MomentaryCost(t)_{JT}} = \sum_{\mathbf{b_{ij}}}^{\mathbf{n}}\left(\mathbf{C_{ij}(t)}\right)$ (eq. 4): **(A)** real data; **(B)** random model and the average $\mathbf{TotalCost(t)_{RJT}}$ (eq. 6): **(C)** real data; **(D)** random model. In the random model, we shuffled the values of the measured speeds for all the bottlenecks in each day and re-assigned them randomly to the bottlenecks. This comparison shows that while the real data fits power law distribution, the random data fits exponential one. Additionally, while the cost of the JTs, based on the real data reach more that 1,000 VH, that of the random model reaches only about a 100 VH. This emphasizes the importance of spatial correlation (which is accounted for in the presented methodology).
